# Highly efficient and genotype-independent barley gene editing based on anther culture

**DOI:** 10.1016/j.xplc.2020.100082

**Published:** 2020-06-05

**Authors:** Yong Han, Sue Broughton, Li Liu, Xiao-Qi Zhang, Jianbin Zeng, Xiaoyan He, Chengdao Li

**Affiliations:** 1 Western Barley Genetics Alliance, College of Science, Health, Engineering and Education, Murdoch University, Murdoch, WA 6150, Australia; 2 Department of Primary Industries and Regional Development, 3 Baron-Hay Court, South Perth, WA 6151, Australia; 3 Western Australian State Agricultural Biotechnology Centre, Murdoch University, Murdoch, WA 6150, Australia; 4 College of Agronomy, Qingdao Agricultural University, Qingdao, Shandong 266109, China

**Keywords:** *Agrobacterium*, CRISPR, genetic transformation, *Hordeum vulgare*, off-target, targeted mutation

## Abstract

Recalcitrance to tissue culture and genetic transformation is the major bottleneck for gene manipulation in crops. In barley, immature embryos of Golden Promise have typically been used as explants for transformation. However, the genotype dependence of this approach limits the genetic modification of commercial varieties. Here, we developed an anther culture-based system that permits the effective creation of transgenic and gene-edited plants from commercial barley varieties. The protocol was tested in Golden Promise and four Australian varieties, which differed in phenology, callus induction, and green plant regeneration responses. *Agrobacterium*-mediated transformation was performed on microspore-derived callus to target the *HvPDS* gene, and T0 albinos with targeted mutations were successfully obtained from commercial varieties. Further editing of three targets was achieved with an average mutation rate of 53% in the five varieties. In 51 analyzed T0 individuals, Cas9 induced a large proportion (69%) of single-base indels and two-base deletions in the target sites, with variable mutation rates among targets and varieties. Both on-target and off-target activities were detected in T1 progenies. Compared with immature embryo protocols, this genotype-independent platform can deliver a high editing efficiency and more regenerant plants within a similar time frame. It shows promise for functional genomics and the application of CRISPR technologies for the precise improvement of commercial varieties.

## Teaser

This study develops an anther culture-based transformation system that permits the efficient creation of transgenic and gene-edited plants from commercial barley varieties. The genotype-independent platform shows promise for genome editing in commercial breeding.

## Introduction

Newly developed gene-targeting and genome-editing techniques facilitate the accurate manipulation of specific genomic sequences, allowing reverse genetics, genome engineering, and targeted transgene integration experiments to be conducted in an efficient and precise manner (Bortesi and Fischer, 2015). As a result, clustered regularly interspaced short palindromic repeats (CRISPR)/CRISPR-associated (Cas) proteins have been discovered and used for precise genetic engineering (Jinek et al., 2012; Jiang et al., 2013; Hsu et al., 2014; Kleinstiver et al., 2016; Chen et al., 2019). Owing to its simplicity of programming and robustness, the CRISPR/Cas9 system is a breakthrough in genome editing, especially for creating targeted mutations to eliminate genes that negatively affect food quality, confer susceptibility to pathogens, or divert metabolic flux away from valuable end products. This tool has provided a plethora of options for genomic engineering in various biological contexts (Mao et al., 2019) and has been applied to all major cereal crops, including wheat (Shan et al., 2013; Wang et al., 2014), rice (Ma et al., 2015; Li et al., 2018), and maize (Liang et al., 2014; Wang et al., 2019). With regard to the regulation and commercialization of CRISPR-edited products, the US Department of Agriculture and the Australian Office of the Gene Technology Regulator have determined that edited crops without foreign DNA are exempt from regulation as genetically modified organisms (Waltz, 2018; Mallapaty, 2019). This decision will enable and promote the use of gene editing technologies for crop breeding to address a changing climate and growing world population.

Barley (*Hordeum vulgare*) is the world’s fourth most important cereal crop. Barley is adaptable, robust, and widely grown across temperate regions worldwide. It has versatile uses as animal feed and in the malting, brewing, and distilling industries. Although barley now forms a minor component of the human diet, it offers potential health benefits as a source of β-glucan and dietary fiber and remains a staple food in several parts of the world, such as Tibet. Barley is a diploid member of the grass family, making it a natural model for the genetics and genomics of the Triticeae tribe. In 2017, a map-based reference sequence of the barley genome, including the first comprehensive, completely ordered 5.3 Gbp assembly, was completed by the International Barley Genome Sequencing Consortium using the North American barley variety Morex (Mascher et al., 2017). More recently, the barley pan-genome project was launched to construct high-quality *de novo* sequence assemblies for a core set of representative genotypes (Monat et al., 2019). The completion of barley pan-genomic sequencing will contribute significantly to gene discovery, genome analysis, and the development and application of genomics-based tools to support barley breeding, including CRISPR/Cas9 genome editing that requires a reference genome to evaluate on-target and off-target activities. The implementation of genome sequencing and physiochemical mutagenesis technologies, together with advances in molecular genetics such as quantitative trait locus (QTL) mapping and genome-wide association studies, have contributed to the identification and functional characterization of vital genes and pathways in barley (Harwood, 2019). Constantly updated information will underpin the future applications of gene editing technologies to the fundamental research and precision breeding of barley.

Gene editing in plants is typically performed by delivering foreign DNA that encodes Cas9 and single-guide RNA (sgRNA) to cultured cells through *Agrobacterium*-mediated transformation. Alternatively, the use of biolistic particle bombardment can facilitate the direct delivery of Cas9-gRNA ribonucleoproteins or *in vitro* transcripts into young embryos to generate transgene-free edited products (Liang et al., 2017). Unlike the convenient transformation of *Arabidopsis* by the floral-dip method, a culture system is required to regenerate plants from explant-derived calli or protoplasts, regardless of the delivery method. However, tissue culture is not always efficient, and culture systems have been developed for only a handful of species and are often optimized for a specific genotype (Mao et al., 2019; Maher et al., 2020). In barley, the genotype dependence of tissue culture protocols is universal and inevitable (Han et al., 2011). The most popular and widely used protocol for barley transformation was optimized for the model cultivar Golden Promise using immature embryos as explants (Tingay et al., 1997; Harwood, 2014). Alternative protocols have been refined for some other barley genotypes, such as the advanced Australian spring barley breeding line WI4330 (Ismagul et al., 2014) and hull-less barley (Lim et al., 2018). Unfortunately, attempts to transform other barley varieties using this procedure have either failed or delivered low transformation frequencies of less than 8% (Hensel et al., 2008). In addition, genetic transformation of androgenetic pollen cultures by *Agrobacterium infection* has been achieved in Igri, the barley variety that responds best to microspore culture (Kumlehn et al., 2006). However, the restricted barley receptors have limited agronomic value and genetic background, causing a bottleneck in the use of gene editing technologies for precise fine-tuning and pyramiding traits of interest in newly released elite commercial varieties.

In the last few decades, anther culture has been used for haploid and/or doubled haploid (DH) production in cereal crops such as maize, wheat, rice, and barley (Ohnoutkova et al., 2019). The induction of plants from meiotic microspores (androgenesis) can cause spontaneous chromosome doubling during the first microspore divisions, resulting in fully fertile DH plants (Castillo et al., 2009). Anther culture response is strongly influenced not only by genotype but also by the growth conditions of the donor plants, the medium composition, and the culture conditions. Although genotype dependence has been observed in barley (Lazaridou et al., 2011), optimized protocols are feasible for a wide range of commercial varieties, breeding lines, and landraces (Broughton et al., 2014), and hundreds of DH populations have been produced from combinations of these germplasms. The developed populations remain a key research tool for genetic mapping and have been widely used to map QTLs for a wide variety of traits in both barley (Watt et al., 2018; Jia et al., 2020) and wheat (Zhang et al., 2018; Choudhury et al., 2019). Moreover, from a molecular breeding perspective, DHs generated by anther culture can segregate gene(s) of interest and speed up breeding programs. Several Australian cereal varieties have been produced by DH breeding, including the barley varieties Dhow, Sloop SA, Flagship, Navigator, Skipper, and Spartacus and the wheat varieties Hume, Gregory, Gladius, Axe, Crusader, Spitfire, Cobra, Gauntlet, Merlin, Fang, and Espada (Broughton et al., 2014).

In this study, we developed a barley genetic transformation and gene editing platform based on anther culture and tested it on a range of commercial varieties. We compared anther culture response among the varieties and performed gene targeting at multiple genomic sites, paying particular attention to mutation rates and off-target activities. Targeted gene mutations were observed in all tested commercial varieties. The platform offers the potential for efficient and precise barley improvement using CRISPR technology.

## Results

### Pipeline for barley anther culture and modification of *Agrobacterium*-mediated transformation

**Figure 1:**
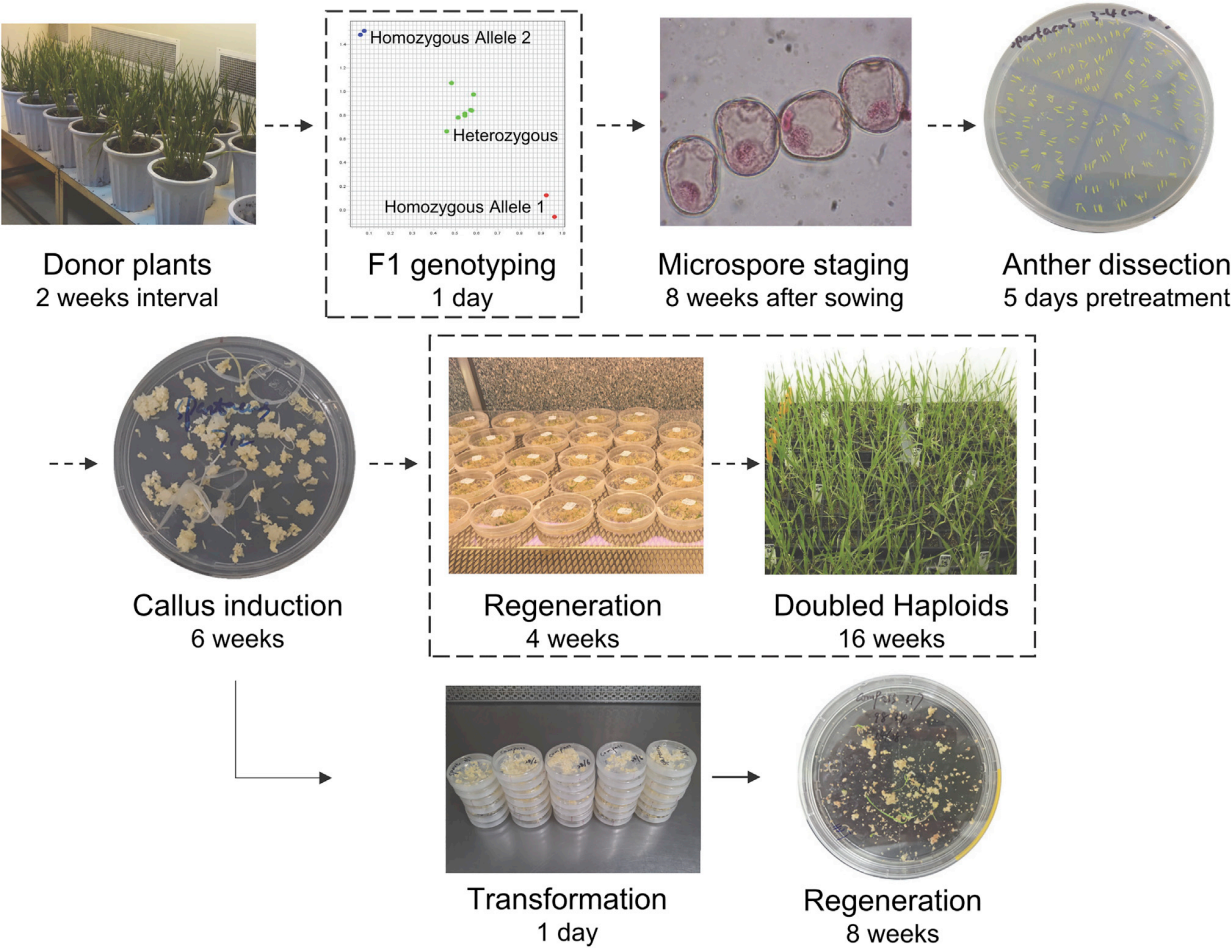
Flowchart of anther culture-based gene editing in barley. The procedures linked by dashed arrows show the typical process of barley anther culture for doubled haploid (DH) production, which takes approximately 35 weeks from the sowing of F1 seeds. The platform is modified for barley genetic transformation and gene editing in commercial varieties, with three steps (framed in boxes) skipped but *Agrobacterium* infection added. After infection, the plantlets regenerate in 6–8 weeks and are suitable for mutant identification. For details, see Methods.

The typical barley anther culture protocol for DH production consists of seven phases: (1) sowing F1 seeds; (2) F1 genotyping for heterozygosity with molecular markers (i.e., KASP); (3) microspore staging to identify the mid- to late-uninucleate stage; (4) anther dissection and mannitol pretreatment (to induce microspore embryogenesis); (5) callus/embryo induction; (6) plant regeneration; and (7) growing regenerants to maturity (Figure 1; Broughton et al., 2014). The whole process takes approximately 10 months from sowing F1 seeds to harvesting the DH lines. We modified the protocol and adapted the platform for *Agrobacterium*-mediated barley transformation and gene editing (Figure 1). First, commercial barley varieties were used as donors, and thus the F1 genotyping step was omitted. Second, after the induction phase, the culture was interrupted after the induction of a mixture of embryos and calli, and only microspore-derived embryogenic calli were transformed with *Agrobacterium* that carried a binary vector for barley CRISPR/Cas9 gene editing (Supplemental Figure 1). The highly efficient method for *Agrobacterium* inoculation meant that up to 100 spikes (or dishes) could be treated per day. Third, a 3-week selection phase was applied, after which viable transformed calli were transferred to a regeneration medium for 5 weeks. Then, green and albino T0 plants were obtained and subjected to transgenic genotyping and mutation identification. We tested the approach with a proof-of-concept experiment that targeted the *HvPDS* reporter gene and subsequently demonstrated targeted gene mutagenesis in multiple commercial varieties.

### Divergent phenology and anther culture responses among commercial barley varieties

**Figure 2:**
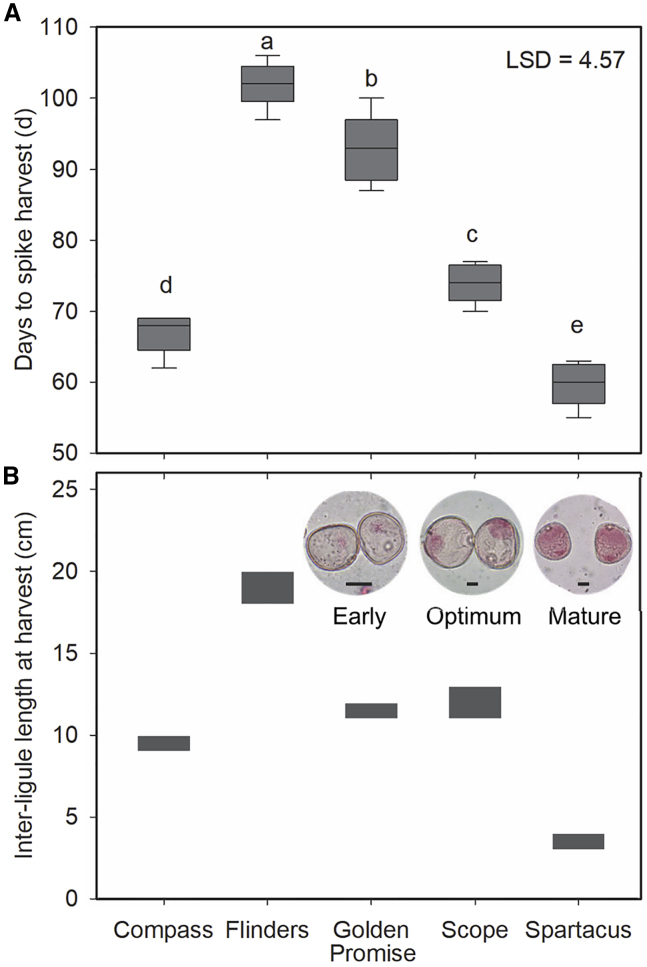
Divergent phenology responses for anther culture in barley. **(A)** Days from seeding to optimum anther dissection time in five commercial varieties. The box plot represents data from five sowing dates in 2017 and 2018. Donor plants were grown in a controlled environment. Different letters indicate significant differences at *p* < 0.05. **(B)** Inter-ligule intervals between the flag leaf and the top-second leaf of tested varieties when microspores reach the optimum late-uninuclear stage. Scale bars, 20 μm.

Four popular Australian commercial barley varieties—Spartacus, Compass, Scope, and Flinders—were developed from different breeding pedigrees in recent years (Supplemental Table 1) and are now the major varieties planted across Australia (https://www.agric.wa.gov.au/barley/2019-barley-variety-sowing-guide-western-australia). When grown in a controlled environment (18°C/13°C [day/night] with a 16-h photoperiod), the Australian varieties, together with Golden Promise, differed significantly in growth and development rates (Figure 2A; *p* < 0.05). Spartacus matured first and, from five sowing dates, required an average of 60 days to reach the mid- and/or late-uninucleate microspore stage, followed by Compass, Scope, and Golden Promise. Flinders took 6 weeks longer than Spartacus to reach the optimum stage for spike harvest. The inter-ligule interval between the flag leaf and the top-second leaf was measured for each variety and correlated with microspore stage (from early to mature), and the appropriate length at which to harvest spikes for anther culture was recorded (Figure 2B). In line with the growth period (Figure 2A), Spartacus spikes were collected with the shortest inter-ligule length of 3–4 cm, whereas Flinders spikes were collected with the longest length of 18–20 cm. Moreover, the growth period and inter-ligule length at harvest were highly correlated in the five varieties (*r* = 0.89, *p* < 0.05).

**Figure 3:**
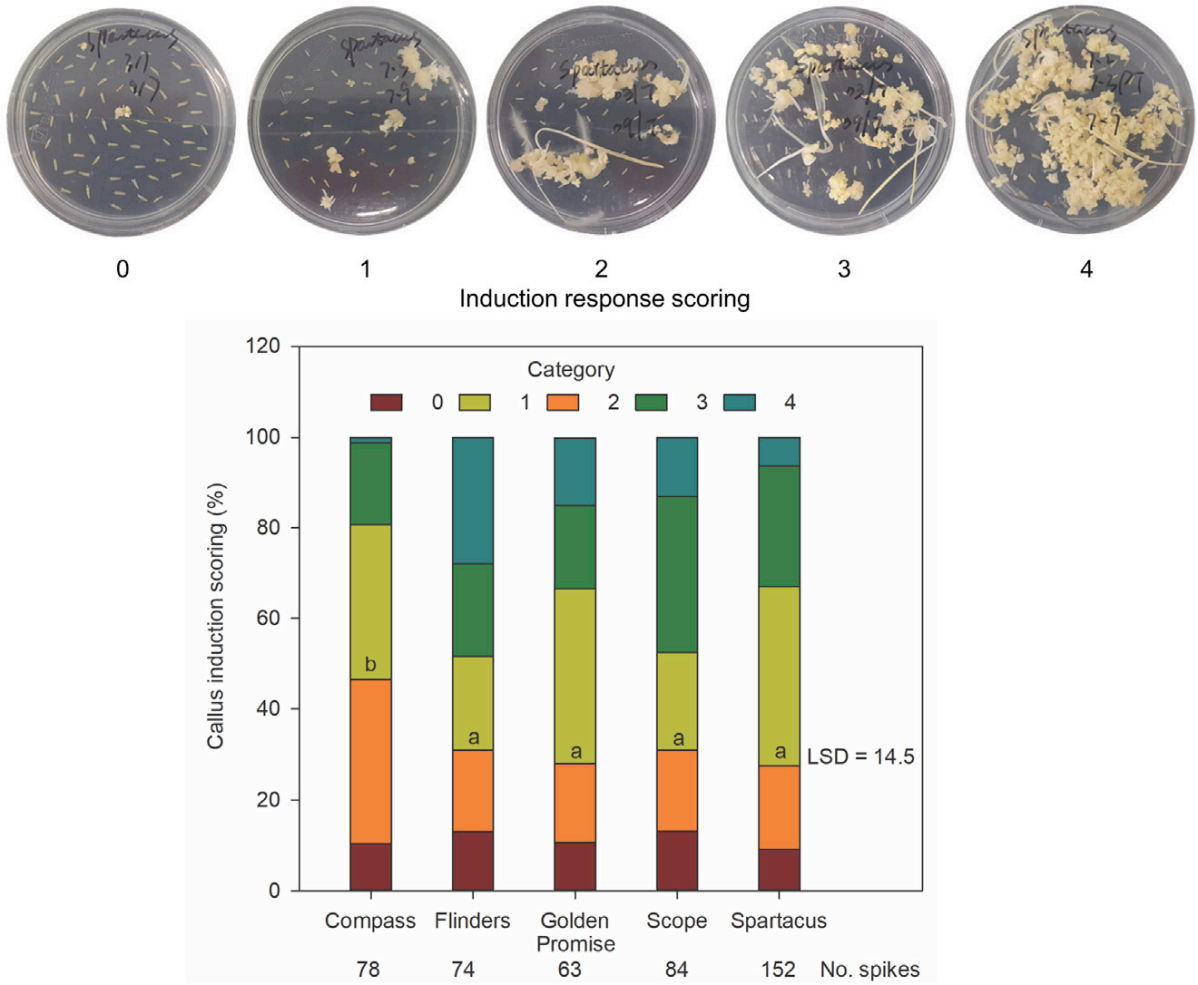
Induction response in five barley varieties. Each Petri dish contained anthers from one spike, and the response was visually scored after 6 weeks. The bar chart represents the average proportions of dishes in each category and comprises all dishes/spikes from two sowing dates in 2018. The number of dissected spikes for each variety is indicated at the bottom of each column. Letters indicate significant differences in the proportion of spikes rated category 2 to 4 in each variety (*p* < 0.10).

**Figure 4:**
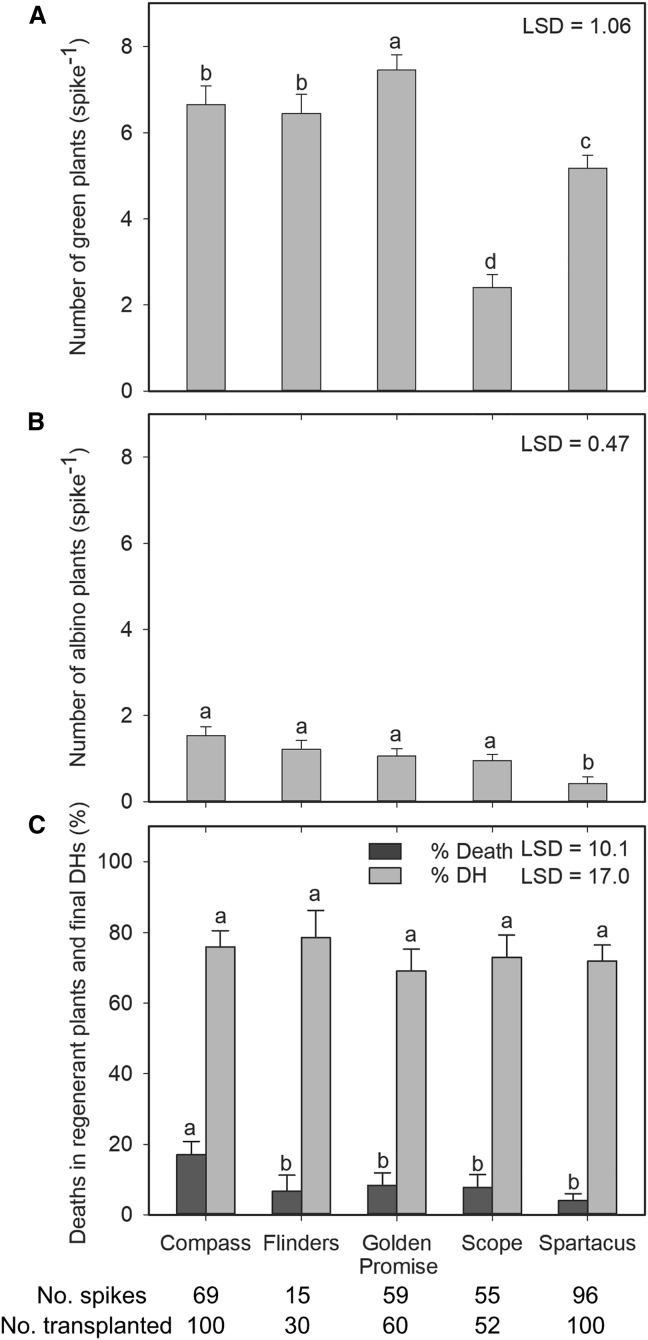
Regenerated green and albino plants from anther culture and chromosome doubling in five barley varieties. **(A and B)** All induced embryos and calli were transferred for regeneration after 6 weeks’ induction, and the numbers of green **(A)** and albino **(B)** plants were counted before transplanting. The number of dissected spikes for variety screening is indicated at the bottom of each column. **(C)** Deaths (%) and chromosome doubling (% DH) in the transplanted plants of five barley varieties. The number of plants transplanted for each variety is indicated at the bottom of each column. Data are presented as means ± SE from three sowing dates in 2017. Different letters indicate significant differences at *p* < 0.05.

After anther dissection and induction, the culture responses and regeneration capability of the five varieties were investigated. As observed in the induction dishes in the transformation experiment, embryo and callus induction differed among varieties but also among spikes within the same variety (Figure 3). The calli from dishes rated category 2 to category 4 performed best and were subsequently selected for *Agrobacterium* infection. In general, the barley varieties shared a similarly high proportion (71%) of spikes rated over category 2; however, Compass had a significantly lower (*p* < 0.1) proportion than the other varieties. In addition, the induction response was not significantly correlated (*p* = 0.76) with green plant regeneration (Figure 4A) in barley, indicating that callus induction is independent of regeneration capability in tissue culture.

Green plant regeneration differed significantly among the five varieties (*p* < 0.001). The number of green regenerant plants produced from each spike (expressed as green plants per spike) ranged from 2.4 in Scope to 7.5 in Golden Promise (Figure 4A). The varieties Compass and Flinders responded similarly to Golden Promise, whereas green plant regeneration was slightly lower in Spartacus. The average number of green plants per spike across all varieties was 5.6. The number of albino plants also differed significantly among varieties (*p* < 0.001). Albino plants per spike ranged from 0.4 to 1.5 (Figure 4B). Overall, there were fewer albino plants than green plants, and the average number of albino plants per spike across all varieties was 1.0.

The number of deaths following transplant differed significantly among the varieties (*p* < 0.048). The highest frequency of deaths was observed in Compass (17%), compared with 7%–8% in most of the other varieties (Figure 4C). The lowest frequency of deaths was recorded in Spartacus (4%). By contrast, the frequency of chromosome doubling (% DH) did not differ significantly among the varieties (*p* = 0.857) and ranged from 69% in Golden Promise to 79% in Flinders (Figure 4C).

When green plants per spike and % DH were combined in a success index of DHs per spike, the results, with the exception of Scope, were relatively consistent among the five varieties (Supplemental Table 2). Scope produced, on average, approximately 1.8 DHs per spike compared with 3.7–5.2 DHs per spike in the other varieties, reflecting the lower green plant regeneration observed in Scope.

### Generation of phenotypic mutants in commercial varieties by targeting *HvPDS*

**Figure 5:**
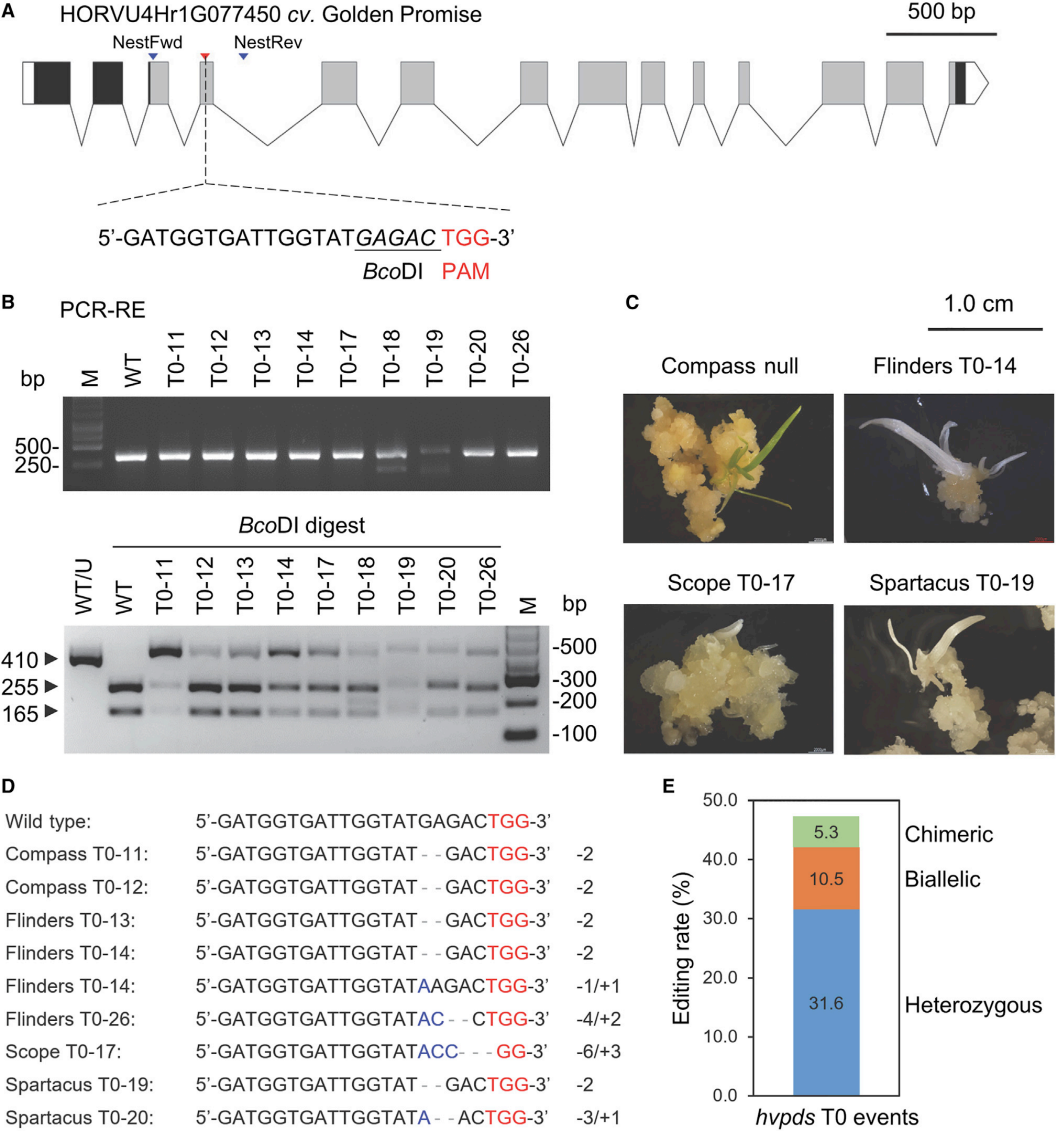
Genetic and phenotypic characterization of the barley *hvpds* mutants. **(A)** Schematic of the *HvPDS* gene in Golden Promise (used as wild type) and the target sequence for gene editing. Boxes and lines represent exons and introns, respectively. The predicted domain of phytoene desaturase is shaded in gray. Scale bar, 500 bp. **(B)** Nested PCR amplification for the gene target and flanking sequence from barley T0 materials, and the restriction digestion of PCR products. **(C and D)** Representatives of regenerated albino mutants **(C)** and gene mutations determined by Sanger sequencing **(D)**. Photos were taken 3 months after anther dissection. **(E)** Editing rates in the T0 events.

Our previous study reported that knockdown of the barley phytoene desaturase gene *HvPDS* by virus-induced gene silencing resulted in a photobleaching phenotype in seedlings (Han et al., 2018). Hence, this phenotypic reporter gene was targeted to test whether genetic transformation and gene mutagenesis could be implemented in the designated platform. The full-length genomic sequence of *HvPDS* was retrieved from the Golden Promise genome, as the gene was not fully sequenced in Morex. *HvPDS* consists of 14 exons and has a predicted phytoene desaturase functional domain (InterPro IPR014102) from AA102 to AA552 (Figure 5A). A 23-bp *G*N_19_*NGG* sequence located on the top strand of the fourth exon was identified and selected to target *HvPDS* via CRISPR/Cas9. The 20-bp target sequence with a 45% GC content was synthesized and assembled into a binary vector for barley gene editing (Supplemental Figure 1). We performed *Agrobacterium*-mediated transformation and, after selection, both green and albino plants were successfully regenerated from the five commercial varieties. T0 materials (proliferated calli and/or plants) with putative hygromycin resistance were randomly sampled for genotyping and mutation identification. Overall, the number of regenerated plants differed among the varieties, with 0.26, 0.42, 0.11, 0.07, and 0.11 per spike from Compass, Flinders, Golden Promise, Scope, and Spartacus, respectively (Supplemental Table 3). Moreover, regeneration efficiency after transformation was not strongly correlated to the performance in the variety screening experiment (*r* = 0.53, *p* = 0.36). The T-DNA backbone was detected in 80% (24/30) of the T0 individuals (Supplemental Table 3), with an editing rate of 47% (9/19) that was subsequently confirmed by a PCR–restriction enzyme (RE) assay with *Bco*DI and Sanger sequencing (Figure 5B and 5D). Both null mutation (green) plants and albinos with targeted mutations in the gene region were obtained from the Australian commercial varieties in the T0 collection (Figure 5C). In the nine edited T0 events, Cas9 from *Streptococcus pyogenes* induced a small insertion/deletion (indel) (<3 bp) and even base substitution within or upstream of the protospacer adjacent motif (PAM) sequence in all commercial varieties except Golden Promise (Figure 5D). A large proportion of the T0 lines were heterozygous (32%), but some mutants carried two or more mutated alleles (Figure 5E). Apart from the induced mutations, Spartacus (T0-19 and T0-20) had a 10-bp insertion within the amplicon, whereas all the other varieties had the same genotype as the Morex barley reference genome (Figure 5B).

### Characterization of on-target and off-target editing in multiple sites of one gene

**Figure 6:**
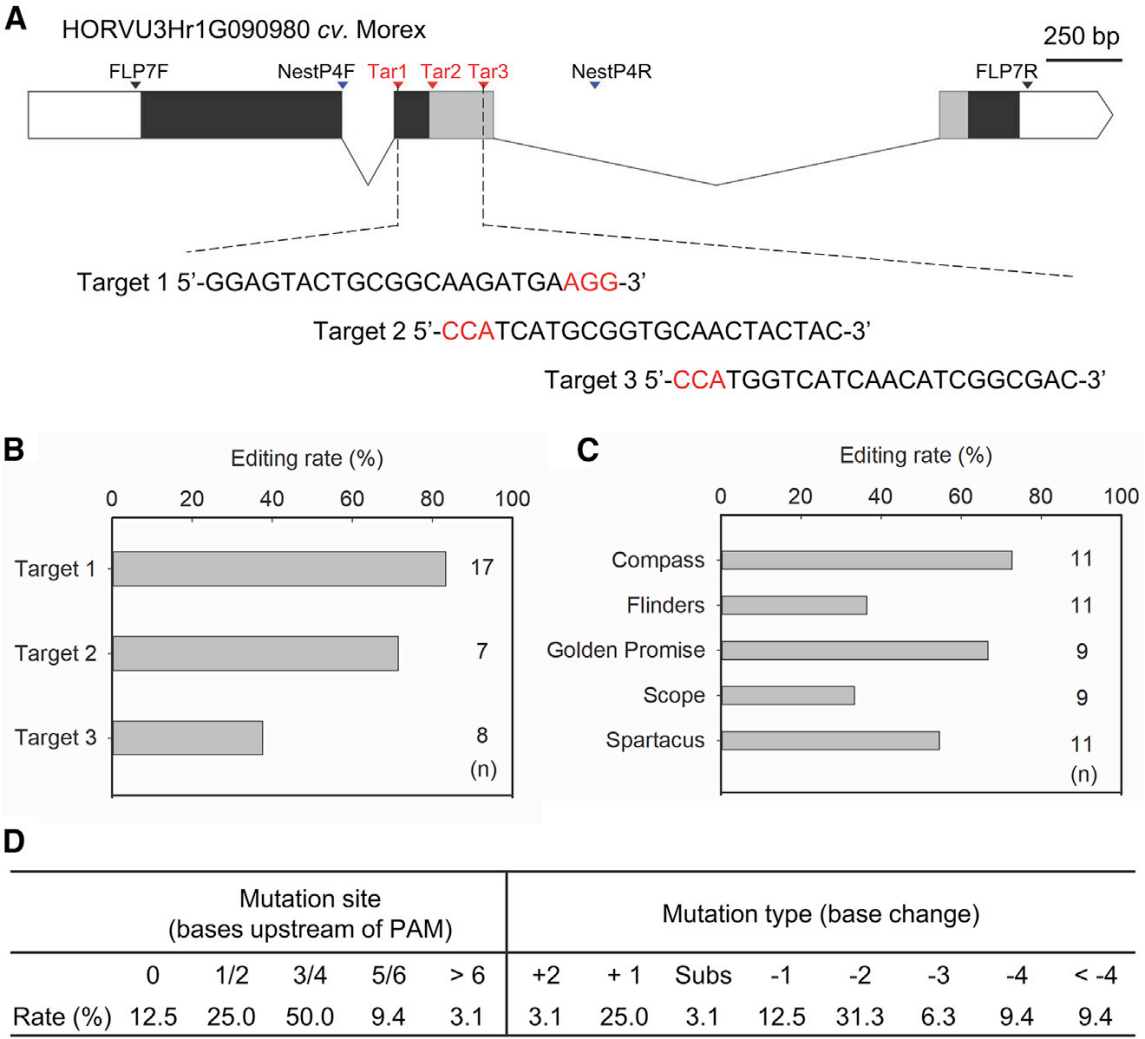
Characterization of targeted gene editing in multiple sites in different barley varieties. **(A)** Schematic of the HORVU3Hr1G090980 gene and the target sequence for gene editing. Boxes and lines represent exons and introns, respectively. The predicted domain of oxoglutarate/iron-dependent dioxygenase is shaded in gray. Scale bar, 250 bp. **(B)** Editing efficiencies of different targeting sites in barley T0 lines. **(C)** Overall editing efficiencies of *HvPDS* and HORVU3Hr1G090980 in different barley varieties. **(D)** Mutation sites and types in *HvPDS* and HORVU3Hr1G090980 editing events. *n*, number of T0 individuals.

Because targeted mutagenesis in *HvPDS* was effective, we characterized gene editing in multiple sites of the barley gene HORVU3Hr1G090980 in different varieties, focusing in particular on editing efficiency and off-targeting activity. Three target sequences from the top or bottom strand of a 300-bp fragment on the second exon were selected, all sharing a GC content of approximately 55% (Figure 6A). Three constructs were assembled and delivered into the barley varieties, and comprehensive genotyping analysis was performed on 32 T0 individuals. Overall, the three target sites had different editing frequencies: Target 1 had the highest rate of 83%, which was 1.2-fold higher than that of Target 3 (Figure 6B). Taken together with the target locus in *HvPDS*, Compass had the highest editing rate of 73% in the four loci, followed by Golden Promise and Spartacus, both with rates over 50% (Figure 6C). By contrast, Flinders and Scope had the lowest editing efficiencies, approximately half that of Compass. A variety of targeted mutations, including the insertion of one or two nucleotides and the deletion of various numbers of nucleotides, were observed in the target regions, and most (50%) occurred 3–4 bp upstream of the PAM sequence (Figure 6D). In the 51 sequenced T0 individuals targeting *HvPDS* and HORVU3Hr1G090980, the original Cas9 generated a large proportion (69%) of single-base indels and two-base deletions in the target sites.

**Figure 7:**
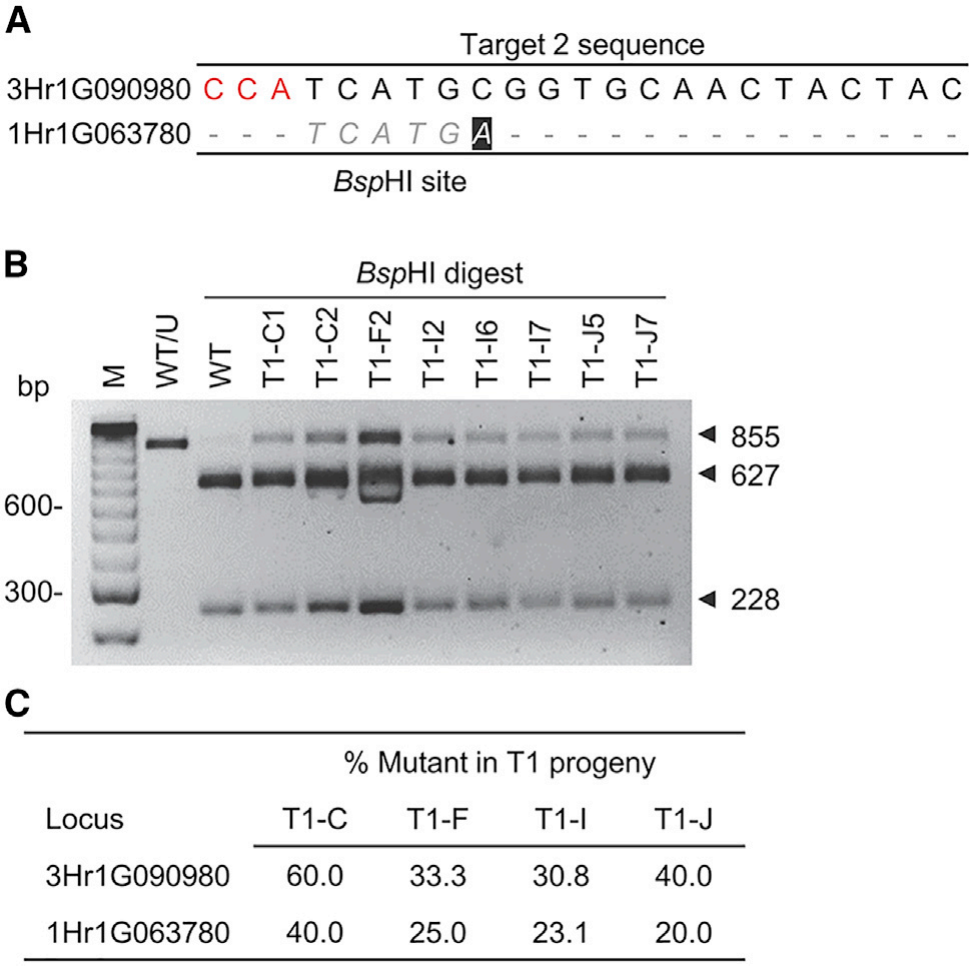
Off-target analysis in barley T1 progenies. **(A)** Alignment of Target 2 sequence with a highly similar hit in the barley Morex genome. The PAM sequence is highlighted in red, and the *Bsp*HI recognition site is in italics. **(B)** PCR–RE assay of eight T1 representatives with off-target mutations. WT, Golden Promise. **(C)** On-target and off-target mutation frequencies in four independent T1 populations.

We also investigated on- and off-target events in T0 and T1 progenies (Figure 7 and Supplemental Figure 2). Target 1 and Target 3 for HORVU3Hr1G090980 had four and one genomic hits with high sequence identities (from 83% to 96%) in the Morex reference genome, respectively (Supplemental Figure 2). However, the 23-bp BLAST hits did not follow the *G*N_19_*NGG* pattern, and no off-target mutations were detected in the 55 T0 individuals of the five varieties. Target 2 had a highly similar hit in the HORVU1Hr1G063780 gene, and a single nucleotide mismatch was located at position 6 of the PAM-proximal region of the sgRNA (Figure 7A and Supplemental Figure 3). The PCR–RE assay and sequencing of four independent T1 Golden Promise progenies indicated that an average of 25% (10/40) of individuals had off-target activity on the HORVU1Hr1G063780 site, and 38% (15/40) of the T1 lines carried the targeted mutations in the designed site for HORVU3Hr1G090980 (Figure 7B and 7C).

## Discussion

Plant tissue culture is a major constraint for transgenic and gene editing studies; current systems have been developed empirically for individual species and are often optimized for a specific genotype (Altpeter et al., 2016). Unlike rice, which has highly efficient *Agrobacterium*-mediated transformation without genotype restrictions for indica, japonica, and javanica cultivars (reviewed by Tyagi and Mohanty, 2000), genotype dependence remains universal and insurmountable in other cultured monocots, including wheat (Delporte et al., 2014), barley (Han et al., 2011), maize (Anami et al., 2010), and sorghum (Mookkan et al., 2017). Most commercially important varieties are recalcitrant or marginally transformable at present. Because culture responses for callus induction and plant regeneration are polygenic, QTL mapping and gene identification for this complex trait have been performed in *Arabidopsis*, rice, maize, barley, and some other crops to identify underlying genetic mechanisms (Bolibok and Rakoczy-Trojanowska, 2006; Chen et al., 2007; Fan et al., 2012; Ikeuchi et al., 2013; Motte et al., 2014; Salvo et al., 2018). The latest breakthrough in genotype dependence is the specific expression of two major transcription factors, *Wuschel2* (*Wus2*) and *Baby Boom* (*Bbm*), from maize, which function as morphogenic regulators that stimulate and promote somatic cells to form embryos that develop into whole plants in a range of monocots, including maize, sorghum, sugarcane, and *indica* rice (Lowe et al., 2016; Mookkan et al., 2017). Most recently, the concomitant expression of developmental regulators and gene editing reagents has generated shoots through *de novo* meristem induction, enabling the rapid production of both transgenic and gene-edited progenies in some dicots, such as tobacco, tomato, potato, and grape (Maher et al., 2020). These procedures could sidestep difficulties in tissue culture and advance crop transformation; however, each tested plant genotype responded differently to the combination(s) of developmental regulators (Lowe et al., 2016; Maher et al., 2020), indicating the need to identify an optimal combination for each genotype. Moreover, 90% of the tested maize inbred lines had a relatively low (<10%) transformation frequency mediated by *Agrobacterium* infection (Lowe et al., 2016), suggesting that crop genotypes are recalcitrant to transformation other than tissue culture and that there are genotypic differences in recalcitrance. In barley, the use of growth-stimulating genes to stimulate morphogenesis has not yet been tested in commercial varieties, and the culture response and efficiency therefore remain unknown. To overcome genotype dependence in tissue culture, we used embryogenic callus derived from microspores rather than scutellar tissues for barley transformation and gene editing (Figure 1), enabling the effective creation of transgenic and gene-edited plants from commercial varieties. The barley varieties tested in this study have diverse genetic backgrounds (Hill et al., 2019; Supplemental Figure 6) and phenology responses (Figure 2), indicating that this relatively genotype-independent system can be adapted for a wide range of commercial varieties. The strategy is also promising for other crop species with established anther culture protocols, such as wheat, rice, and oats (edited by Touraev et al., 2009).

Chromosome doubling is a vital step in the production of fertile DH plants during anther culture; it can either occur spontaneously or be induced by chemical treatment. We observed high frequencies of spontaneous chromosome doubling in the plants that survived to maturity, ranging from 69% to 79% in the five barley varieties (Figure 4), consistent with previous findings (Castillo et al., 2009; Broughton et al., 2014). In other crops, spontaneous chromosome doubling is much more variable and highly genotype-dependent, with frequencies of 0%–77% in cabbage (Yuan et al., 2015), 43%–88% in broccoli, and 7%–91% in other coles (Da Silva Dias, 2003). Variations in the frequency of spontaneous chromosome doubling in monocot species range from 24% to 80% in bread wheat (Broughton et al., 2014), 6% to 38% in triticale (Würschum et al., 2012), and 0.4% to 70% in maize (Chaikam et al., 2019). The high, stable chromosome doubling frequency of barley makes it a promising species for anther culture and anther culture-based gene editing. In addition, the anther culture protocol described here delivered relatively robust results across a range of genotypes. Although significant differences in green plant regeneration were observed, green plants were successfully produced from all varieties, and the observed genotypic variation was consistent with other reports (Kasha et al., 2001; Makowska et al., 2015). Although the genetic control of androgenesis has not been fully characterized in barley, the current protocol is relatively genotype-independent for induction and regeneration responses. It has been reported that mannitol pretreated barley microspores can result in fused nuclei, resulting in DH microspores and high rates of chromosome doubling. Such pretreatment provides genotype-independent induction and suspension of nuclear division (Kasha et al., 2001), which appears to be a possible cause of the low genotype dependence in barley.

The production of albino plants was observed in this study, albeit at low frequencies. Although this can be a problem following androgenesis in some species and cultivars, it was not an issue in the present study. The direct cause of albinism in androgenesis-derived plants is the inability of proplastids to transform into chloroplasts, and this trait is influenced by both genetic and environmental factors (Kumari et al., 2009; Makowska and Oleszczuk, 2014). The mannitol pretreatment used to initiate embryogenesis in the current study also promoted green plant regeneration in large numbers of barley crosses (Cistué et al., 1994; Broughton et al., 2014) and may help to reduce albino plant numbers.

Interestingly, the mutation types induced by the CRISPR/Cas9 system in the first transgenic generation in this study differ from those in other reports on barley (Kapusi et al., 2017; Gasparis et al., 2018). Most of the biallelic and heterozygous mutations (67%) at the four target sites were generated in the T0 barley with this system (Figures 5 and 6), whereas in the two previous studies, most of the tested plants appeared to be chimeric with three or more mutations. A similar pattern in the proportion of mutations was found between the first transgenic generation of rice and *Arabidopsis* (Ma et al., 2015), with more uniform mutation types detected in T0 rice and more heterozygous and chimeric mutations in *Arabidopsis*. Because different explants and transformation methods were used in these studies, targeted editing mainly occurred in transformed callus cells before regeneration (in this study and rice). By contrast, editing events can occur early, as in *Arabidopsis* ovules and zygotes, or later in vegetative tissues (Ma et al., 2015). Likewise, induced mutations in barley may be generated in the cells of embryos, callus, and regenerated plants during tissue culture (Lawrenson et al., 2015) when immature embryos are used as explants. Notably, the established anther culture system not only serves as a toolkit for barley gene editing but also for segregating the T-DNA backbone and desired mutations in early generations. Combining anther culture and marker-assisted selection could generate homozygous DH plants with mutations of interest and thereby accelerate the breeding program.

In addition to the use of plant codon-optimized *Cas9*, plant CRISPR/Cas9-induced editing efficiency is also determined by the transcript levels of *Cas9* and sgRNA, and by the sequence features of the targets. In barley, the *Ubiquitin* promoter is often used to drive constitutive *Cas9* expression, and the sgRNA cassette is driven by U6 promoters from wheat (Lawrenson et al., 2015; Gasparis et al., 2018; and this study) or rice (Kapusi et al., 2017). Although different explant types were used for barley tissue culture (anthers versus immature embryos), the high editing rate in Golden Promise (67%) was consistent with that reported in two recent studies (Kapusi et al., 2017; Gasparis et al., 2018) but much higher than the first reported frequency of 10%–23% (Lawrenson et al., 2015). The system demonstrated that high editing rates are possible in other commercial varieties, although there were remarkable differences in efficiency among varieties (Figure 6C). The infected callus often developed necrosis and turned brown, such that Flinders and Scope had low editing rates, suggesting that they are more susceptible to *Agrobacterium* infection. Therefore, the strength of *Agrobacterium* inoculation and the duration of co-cultivation with calli must be further optimized for such varieties.

The variable cleavage efficiencies among different targets confirmed in this study (Figure 6B) are consistent with previous observations in other cereal crops, including rice (Ma et al., 2015) and wheat (Liang et al., 2017). The cleavage efficiency is highly dependent on the selected target sequence (i.e., GC content, specificity in the host genome), which strongly correlates with target–sgRNA folding stability. Interestingly, the efficiencies of the three targets are not in line with their predicted rates in CRISPOR (see Methods), in which their similar predicted scores range from 54 to 64. Although the GC contents of the three target sites are equal, their PAM sequences are different. It is difficult to determine whether the PAM sequence determines the rate (Target 1 with an AGG PAM is more efficient than Targets 2 and 3 with a TGG PAM), as the number of guides is limited, and targets with the same TGG PAM also varied in efficiency in the present study. Therefore, the PAM sequence is a possible cause of efficiency variation, but other unknown factors may be involved. It is noteworthy that mainly single-base indels and two-base deletions at target sites were detected within 4 nt upstream of the PAM (Figure 6D), consistent with previous reports in rice (Ma et al., 2015), barley (Kapusi et al., 2017; Gasparis et al., 2018), and wheat (Liang et al., 2017). It has been well documented that Cas9 induces DNA double-strand breaks (DSBs) and that the DSBs are repaired by the error-prone non-homologous end-joining pathway, which typically introduces 1- to 4-bp indels due to the annealing of single strands with short regions of microhomology (Lieber, 2010; Jinek et al., 2012; Hsu et al., 2014). The introduced indels lead to a frameshift in the targeted gene and have the potential to generate knockout lines for functional characterization and breeding.

We investigated the off-target genes predicted by the CGAT and CRISPOR tools, and we intentionally selected Target 2 to validate off-targeting in practice. We did find off-target mutations in the target DNA that had single-nucleotide polymorphisms with guide RNA (Figure 7), similar to those reported in another barley editing investigation (Lawrenson et al., 2015). It is noteworthy that off-targeting could be elusive from sites with highly similar sequences but without the *G*N_19_*NGG* structure (Supplemental Figure 2). Whole-genome sequencing has revealed that off-target mutations are even rarer than inherent genetic and/or somaclonal variations in CRISPR/Cas9-edited plants (Li et al., 2019), and negative mutations that affect the phenotype of interest can be segregated during sexual reproduction (Mao et al., 2019). Nonetheless, our results indicate that appropriate target sites must be selected by genome searching in order to minimize undesired off-target mutations (Xie et al., 2014; see Methods), as the sgRNA tolerates a certain number of mismatches between the guide RNA and the target DNA (Hsu et al., 2013).

Typical barley transformation using immature embryos as explants requires up to 28 weeks from sowing donor plants to first-generation regenerants (15 weeks until embryo harvest and 13 weeks for culture). Such protocols generate approximately 0.03 transgenic plants per embryo and deliver a gene editing rate of 65% in Golden Promise (Kapusi et al., 2017; Gasparis et al., 2018). With anther culture, the whole process can be accomplished in a similar time frame (13 weeks to spike harvest and 14 weeks for culture), but with a higher throughput of 0.08 plants per spike and a comparably high editing rate of 67%. In addition, the DH platform can segregate the foreign vector backbone and targeted mutations in the early generation, thereby generating transgene-free homozygous DH plants with mutations of interest. In summary, we developed a genotype-independent platform for highly efficient genetic transformation and gene editing in commercial barley varieties. The system shows promise for other crop species with established anther culture protocols and has the potential to promote the implementation of CRISPR technologies for functional genomics research and the precise genetic improvements of commercial varieties.

## Methods

### Barley varieties and growth conditions

Four Australian commercial malting barley varieties, Compass, Flinders, Scope, and Spartacus, and the barley transformation reference Golden Promise, which was released in the United Kingdom in 1968, were used in this study. Donor plants for anther culture were grown as described in Broughton et al. (2014), with plants maintained in a controlled environment at 18°C/13°C (day/night) with a 12-h photoperiod.

### Target locus, single-guide RNA design, and plasmid construction

The genomic sequence of the barley *HvPDS* gene (gene ID HORVU4Hr1G077450) was obtained from the Golden Promise Genome blast server (https://ics.hutton.ac.uk/gmapper/gmap_page.html) at the James Hutton Institute, UK. The gene sequence of HORVU3Hr1G090980 (which is annotated as *Gibberellin 20-oxidase 3* and is the candidate for the *sdw* locus responsible for semi-dwarfism in barley; Xu et al., 2017) was retrieved from the barley Morex reference genome using the IPK Barley BLAST Server (https://webblast.ipk-gatersleben.de/barley_ibsc/). Gene structure and annotation were predicted by EnsemblPlants (http://plants.ensembl.org/index.html), and schematic diagrams for both genes were generated with Exon-Intron Graphic Maker (http://wormweb.org/exonintron). Preliminary target sequences were identified using http://cbc.gdcb.iastate.edu/cgat/and CRISPOR (http://crispor.tefor.net/), both of which have the barley reference genome for off-target evaluation. Potential targets were further filtered for a 23-bp *G*N_19_*NGG* sequence because the sgRNA would be driven by the U6 promoter and coupled with the Cas9 nuclease derived from *S*. *pyogenes* that recognizes a PAM sequence of NGG. The final target sequences were selected based on specificity in the whole genome to minimize off-target activity and optimize GC content, distance from the start codon, and putative editing efficiency. The four selected guides were 5′-GAT GGT GAT TGG TAT GAG ACT GG-3′ targeting *HvPDS* and 5′-GGA GTA CTG CGG CAA GAT GAA GG-3′ (Target 1), 5′-CCA TCA TGC GGT GCA ACT ACT AC-3′ (Target 2), and 5′-CCA TGG TCA TCA ACA TCG GCG AC-3′ (Target 3) targeting HORVU3Hr1G090980. PCR and Sanger sequencing were performed to confirm that no sequence polymorphism was present in the targets of the five selected barley varieties.

A binary vector (pBarge, Supplemental Figure 1A) that housed a hygromycin resistance cassette (Addgene #68263), a Cas9 expression cassette (Addgene #68258), and an sgRNA was assembled for barley genome editing, following the protocol described in Lawrenson et al. (2015) using the Golden Gate cloning system (Weber et al., 2011). First, Level 1 assembly was performed to construct the gRNA gene that targeted *HvPDS*. After verification with restriction digestion by *Apa*LI (New England Biolabs, Australia), Level M assembly was performed to construct the final binary vector for delivery into barley. The linkers, receptors, and plasmid backbones were available in the MoClo Plant Parts Kit (Addgene #1000000047) and the MoClo Toolkit (Addgene #1000000044) described in Engler et al. (2014). To construct a new pBarge vector that targeted a specific genomic locus, we modified the Golden Gate assembly strategy to Restriction and Insertion cloning (named FastTrack, Supplemental Figure 1B) to reduce the two assembly steps to one. Specifically, a first-round PCR was performed to amplify a 422-bp product from any constructed pBarge vector; the amplicon was then used as a template for the second-round PCR to synthesize a 70-mer forward primer that contained a new target sequence (Integrated DNA Technologies, Australia) to be used with the same reverse primer in the first-round PCR. Both the 479-bp amplicon and any constructed pBarge plasmid DNA were double digested with two restriction enzymes, *Age*I and *Not*I (New England Biolabs, Australia). After digestion, the products were purified and ligated using T4 ligase (New England Biolabs, Australia) before being transformed into *Escherichia coli* competent cells (Top10 strain). White colonies were selected, and the fidelity of the clone was confirmed by restriction digestion (*Apa*LI) and sequencing. Eventually, the target sequence in the original pBarge vector was replaced by the target of interest. Three sgRNAs that targeted HORVU3Hr1G090980 were designed and assembled separately into the pBarge vector using FastTrack cloning as described. The primers used for molecular cloning are listed in Supplemental Table 4. Plasmid DNA was extracted using the ISOLATE II Plasmid Mini Kit (Bioline, Australia) and then transformed into *Agrobacterium tumefaciens* strain AGL1 via electroporation (Bio-Rad Gene Pulser II System, USA).

### Anther culture variety screening

The barley anther culture protocol is described in Broughton et al. (2014), with modifications for *Agrobacterium* infection and genetic transformation (Figure 1). Donor plants were grown to the early booting stage (Zadoks scale Z41) with the flag leaf sheath extending (Zadoks et al., 1974). As each variety reached this stage, the inter-ligule interval between the flag leaf and the top-second leaf was measured so that the distance could be correlated with the microspore stage for subsequent spike harvest (Esteves and Belzile, 2019). Spikes were then removed from the leaf sheaths, and the microspore stage was determined by squashing the anthers in 2% acetocarmine stain (w/v) and examining the microspores under 400× magnification using an optical microscope (Olympus BH2, Japan). Spikes with microspores at the mid- to late-uninucleate stage were selected for anther culture. Spikes were harvested daily over 2 weeks (3 weeks for Flinders) and stored at 4°C in a beaker of water, then sterilized and processed in batches. The number of spikes harvested for each variety varied, as some varieties produced more spikes, and the number of batches varied accordingly. The number of spikes/batches for each variety was as follows: Compass (69/2), Flinders (15/2), Golden Promise (59/3), Scope (55/4), and Spartacus (96/4). Following spike sterilization, between 60 and 80 anthers were removed from each spike for mannitol pretreatment in a 55 × 14-mm Petri dish that contained 5.9 g l^−1^ CaCl_2_·H_2_O, 182 g l^−1^ mannitol, and 20 g l^−1^ agar (Sigma-Aldrich #A7921). Dishes were then sealed with Parafilm and incubated in darkness at 25°C for 5 days. After the 5-day pretreatment, anthers from each spike/dish were transferred to the induction medium (Broughton et al., 2014) in a 55 × 14-mm Petri dish. Dishes were sealed and incubated in the dark at 25°C for 5–6 weeks. Microspore-derived embryos and calli were then transferred to regeneration medium in 90 × 20-mm Petri dishes and incubated in a culture room (25°C, 16-h photoperiod) for 4 weeks to compare the regeneration capability of the five varieties. The numbers of green and albino plants in each Petri dish (each dish represented one spike) were counted. Subsets of green plants from each variety (30–100) were transplanted into 20-cell seedling trays that contained a potting mix, and the plants were grown to maturity in a greenhouse. Deaths were recorded, and surviving plants were scored for ploidy/fertility at harvest. Ploidy was determined visually; plants with seeds were classed as DHs, whereas sterile plants were classed as haploids.

### Transformation

Barley transformation was performed using the anther culture protocol described above with modifications for *Agrobacterium* infection and genetic transformation (Figure 1). For the transformation experiment, anther culture was performed on a parallel set of spikes (Figure 3) and interrupted after induction, with microspore-derived embryogenic calli used as receptors for *Agrobacterium* infection (Figure 1). Induction responses were scored (0–4) for each dish/variety combination, and representative dishes were photographed. All plants and embryos with radicles were then removed from the Petri dishes. *Agrobacterium* culture was prepared 1 day before infection in 15 ml of liquid MG/L medium with appropriate antibiotics and finally diluted with fresh medium to an OD_600_ of 0.6 (PerkinElmer Lambda 25 UV/VIS Spectrophotometer, USA) for infection. For each construct, a spray trigger (Canyon CHS-3AN, Australia) was first adjusted to an even stroke of 0.75 ml per spray and then surface-sterilized with 20% sodium hypochlorite (12.5% [w/v] stock solution from Rowe Scientific, Australia), rinsed with sterile water, and air-dried in laminar flow. Nine dishes (55 × 14 mm) were placed on a piece of glass with lids off and sprayed with four strokes of *Agrobacterium* cell mist, one from each direction. The dishes were then stacked, wrapped with cling film and aluminum foil, and kept in darkness at 25°C for 3 days of co-cultivation. The calli were subsequently transferred for selection on B13M medium that contained the salts and vitamins described in Han et al. (2011), as well as 750 mg l^−1^ L-glutamine, 100 mg l^−1^ myoinositol, 2.5 mg l^−1^ 2,4-dichlorophenoxyacetic acid, 0.1 mg l^−1^ 6-benzylaminopurine, 1.25 mg l^−1^ CuSO_4_·5H_2_O, 3.5 g l^−1^ Phytagel, 160 mg l^−1^ Timentin, and 30 mg l^−1^ hygromycin, with the final pH adjusted to 6.0. After 3 weeks, resistant calli that showed a flaxen color were removed for a second selection in deep Petri dishes (90 × 20 mm) under low-light conditions (Harwood, 2014). After 2 weeks, the proliferated calli with green buds and/or plumules were transferred to R medium (Harwood, 2014) in deep dishes. Plants were photographed using a stereomicroscope (Nikon SMZ18, Japan) and finally transferred to growth tubs (60 × 100 mm) that contained regeneration medium with Murashige and Skoog basal salts, 20 g l^−1^ maltose, 120 mg l^−1^ Timentin, 30 mg l^−1^ hygromycin, and 3.0 g l^−1^ Phytagel (pH 6.0).

### Genotyping of barley callus and plants

Barley genomic DNA was extracted using the cetyltrimethylammonium bromide method (Murray and Thompson, 1980). Primers were designed based on the barley reference genome sequence using Primer-BLAST (https://www.ncbi.nlm.nih.gov/tools/primer-blast). LA Taq (Takara, Japan) and BIOTAQ DNA polymerase (Bioline, Australia) were used for long fragment and normal PCR amplification, respectively, following the manufacturers’ instructions. Sanger sequencing was performed at the Western Australian State Agricultural Biotechnology Center, Murdoch University. Some lines with heterozygous and biallelic mutations were cloned into the pGEM-T Easy vector (Promega, Australia), and the colonies were then sequenced. PCR–RE assays for the *HvPDS* gene and off-target mutations were performed with *Bco*DI and *Bsp*HI (New England Biolabs, Australia), respectively. Primers for gene cloning, nested PCR, and sequencing are listed in Supplemental Table 4.

### Statistics

The number of green plants per spike and the number of albino plants per spike were analyzed with linear mixed models in R (R Core Team, 2019) using ASReml-R version 4.1.0 (Butler et al., 2018). The fixed effect in all models was variety, and the random effect was batch number.

The percentage (proportion) of dead and DH plants was analyzed using Genstat Edition 19 (http://genstat.com). The HGLM (hierarchical generalized linear model) procedure was used to fit a generalized linear model for these traits. The percentage of dead plants was calculated as (number of plants that died/total number of plants transplanted) × 100. The percentage of DH plants was calculated as (total number of DH plants/number of transplanted plants that survived to maturity) × 100. Both traits were analyzed using a binomial model with the identity link function, with variety as a fixed effect.

Treatment means were compared using 5% least significant differences (LSDs). LSDs were calculated by multiplying the average SE of difference by 2 (5% LSD).

Pearson's correlation coefficient between traits was estimated using the IBM SPSS Statistics Package (Version 24).

## Funding

This research was supported by the 10.13039/501100011024Western Australian Department of Primary Industries and Regional Development and the Western Australian State Agricultural Biotechnology Center, 10.13039/501100001799Murdoch University.

## Author Contributions

Y.H. and C.L. conceived and designed the research. S.B. and L.L. grew all donor plants and instructed Y.H., X.-Q.Z., J.Z., and X.H. to perform tissue culture. Y.H. and S.B. analyzed the data. Y.H. wrote the manuscript with input from all authors.

## Supplemental Information

Document S1. Supplemental Figures 1–3 and Supplemental Tables 1–4

Document S2. Article plus Supplemental Information
