# Supplementary material for: Highly efficient and genotype-independent barley gene editing based on anther culture
Source: Plant Commun. 2020 Jun 5;2(2):100082. doi: 10.1016/j.xplc.2020.100082 (PMC8060703; doi:10.1016/j.xplc.2020.100082)
Supplement: Document S1. Supplemental Figures 1–3 and Supplemental Tables 1–4 [file mmc1.pdf]

Plant Communications, Volume *2*

Supplemental information

Highly efficient and genotype-independent barley gene editing based on anther culture

Yong Han, Sue Broughton, Li Liu, Xiao-Qi Zhang, Jianbin Zeng, Xiaoyan He, and Chengdao Li

Supplemental Information

Highly efficient and genotype-independent barley gene-editing based on anther culture

Yong Han[1,3], Sue Broughton[2], Li Liu[2], Xiao-Qi Zhang[1,3], Jianbin Zeng[4], Xiaoyan He[4], Chengdao Li[1,2,3]

[1] Western Barley Genetics Alliance, College of Science, Health, Engineering and Education, Murdoch University, WA 6150, Australia

[2] Department of Primary Industries and Regional Development, 3 Baron-Hay Court, South Perth, WA 6151, Australia

[3] Western Australian State Agricultural Biotechnology Centre, Murdoch University, WA 6150, Australia

[4] College of Agronomy, Qingdao Agricultural University, Qingdao, Shandong 266109, China

Correspondence: c.li@murdoch.edu.au

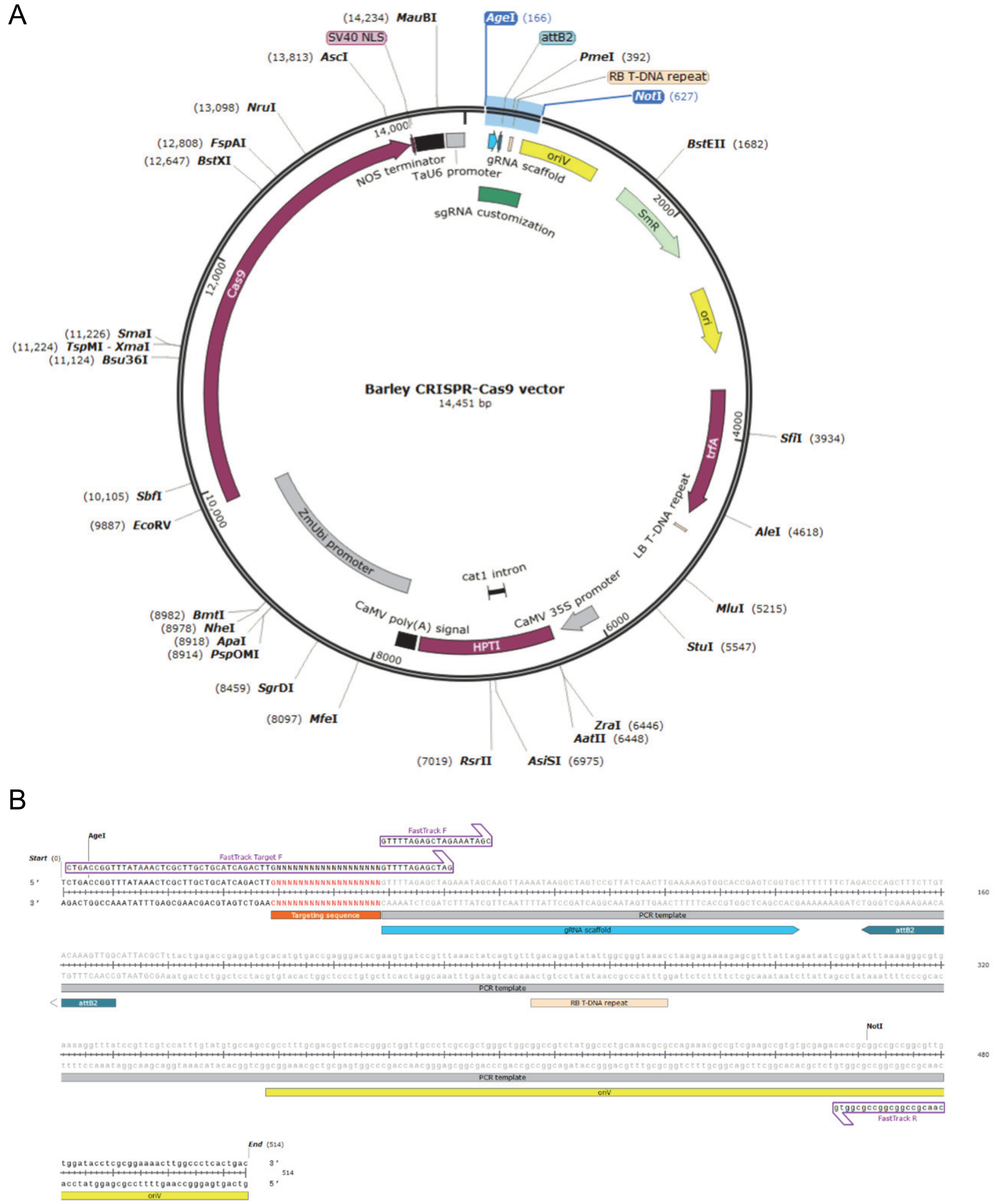

Supplemental Figure 1 pBarge plasmid map and the FastTrack construction strategy. (A) The binary vector containing the *Cas9*, *HPT* and sgRNA expression cassette for barley gene-editing. (B) Cloning strategy for new genomic targets. See Methods for details.

	Target 1 sequence	Identity (%)	T0 off-target
3Hr1G090980	G G A G T A C T G C G G C A A G A T G A A G G		
3Hr1G113450	- **T**	95.6	0/12
5Hr1G070000	**C** - - - - - - - - - - - **G** - - - **G** - - - - - **A**	82.6	0/12
5Hr1G114260	- - - - **G** - - - - - - - - - - - - **A** - - **G** - **C**	82.6	0/12
6Hr1G005780	- - - - - - - - - - - **A** - **G** - - **G** - - - - - **A**	82.6	0/12

	Target 3 sequence	Identity (%)	T0 off-target
3Hr1G090980	C C A T G G T C A T C A A C A T C G G C G A C		
2Hr1G093950	- **G** **T** - - - - - - - - - - - - - - - - - - - **T**	87.0	0/7

Supplemental Figure 2 Off-target analysis for genomic regions with high sequence identities in barley T0 progenies.

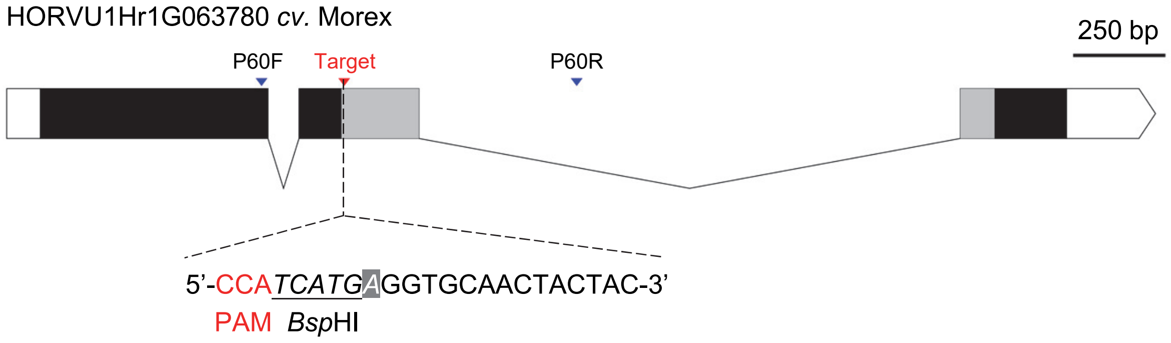

Supplemental Figure 3 Schematic of barley 1Hr1G063780 gene and the off-target hit by 3Hr1G090980 Target 2 with one single SNP. Boxes and lines represent exons and introns, respectively. Predicted domain of the Oxoglutarate/iron-dependent dioxygenase and the SNP are shaded in grey. Scale, 250 bp.

Supplemental Table 1 Biography of varieties used in the study

Variety Name	Pedigree	Granted	Classification	Title Holder	Detailed Description
Compass	Commander x F1 (County x Commander)	2015	Malting	Adelaide Research & Innovation Pty Ltd and GRDC	Click Link
Flinders	Baudin x Cooper	2017	Malting	InterGrain Pty Ltd	Click Link
Golden Promise	γ-ray mutation from Maythorpe	1968	Malting	Miln Marsters	Click Link
Scope	EMS mutation from Buloke (Franklin x VB9104)	2011	Malting	Agriculture Victoria Services Pty Ltd	Click Link
Spartacus	Scope x 4*Hindmarsh	2018	Malting	Intergrain Pty Ltd and Agriculture Victoria Services Pty Ltd	Click Link

Supplemental Table 2 Success index, expressed as DHs per spike, in five barley varieties.

Variety	Green plants per spike	% DH	DHs per spike
Compass	6.7	76	5.1
Flinders	6.5	79	5.1
Golden Promise	7.5	69	5.2
Scope	2.4	73	1.8
Spartacus	5.2	72	3.7

Supplemental Table 3 Summary of culture performance and mutation rate via targeting *HvPDS*

Variety	Number of spikes	Number of T0 plants	T0 plants per spike	Samples for genotyping	Samples with T-DNA	Mutation rate
Compass	42	11	0.26	6	5	2/4
Flinders	50	21	0.42	8	6	3/5
Golden Promise	45	5	0.11	4	2	0/2
Scope	58	4	0.07	6*	5	2/4
Spartacus	111	12	0.11	6	6	2/4
Total/Average	306	53	0.19	30	24	9/19

* Two pieces of proliferated calli were sampled for DNA extraction and genotyping. Spikes were harvested from donor plants sown in two different dates in 2018.

Supplemental Table 4 Primers for gene cloning, plasmid construction, nested-PCR and Sanger sequencing.

Purpose	Name	Sequence (5' – 3')
Gene cloning	*HvPDS* P53F	TACGCAGAGGTGCTTCACAA
	HvPDS P48R	CGGAGATCAGTTTGTACTTCA
	3Hr1G090980 FLP7F	CTCACACACCTCTCATCTCATGG
	3Hr1G090980 FLP7R	GAATCAGCCCGTGGATGGAG
	1Hr1G063780 P60F	CGACTTCATGCACCTAGGGTT
	1Hr1G063780 P60R	ATGCAGCTCGTTCGATCGTT
	$35S_{Pro}$ detection F	GCTCCTACAAATGCCATCATTGC
	$35S_{Pro}$ detection R	GATAGTGGGATTGTGCGTCATCCC
	Cas9 detection F	CTACGCCGGATACATTGACGG
	Cas9 detection R	GATTTGCGAGTCATCCACGCG
	3Hr1G113450 P38F	ACGGTTTGGCCAGGATGAAT
	3Hr1G113450 P38R	CAGGGTTATGCGTGCCAATG
	3Hr1G113450 P63F (Scope)	AAAGCCAGCACTCATGGACA
	3Hr1G113450 P63R (Scope)	GCGGCAAATTGGTTGAAGGT
	5Hr1G070000 P40F	GCACCAGCTTGTTTCAGCAC
	5Hr1G070000 P40R	AGGATAATGCTACGCCGATGA
	5Hr1G114260 P42F	GTTCCGCGGGTTCACGA
	5Hr1G114260 P42R	CCATCAGCGCGAATTGTGGCA
	1G005780 P43F	GGAATCATCCACACCGTCGT
	1G005780 P43R	CACCACCAACAACGTGATCG
	2Hr1G093950 P62F	CGCTGGGTCGCCGTCAACC
	2Hr1G093950 P46R	CTTCCCGTATGGCGACACAT
Plasmid construction	sgRNA for *HvPDS*	tgtggtctcaCTT GATGGTGATTGGTATGAGAC gttttagagctagaaatagcaag
	sgRNA Reverse	tgtggtctcaAGCGtaatgccaactttgtac

Supplemental Table 4 (*continued*)		
Plasmid construction	FastTrack 1st PCR F	GTTTTAGAGCTAGAAATAGC
	FastTrack 1st PCR R	CAACGCCGGCGGCCGCGGTG
	FastTrack 3Hr1G090980 Tar1	CTGACCGGTTTATAAACTCGCTTGCTGCATCAGACTTGGAGTACTGCGGCAAGATGAGTTTTAGAGCTAG
	FastTrack 3Hr1G090980 Tar2	CTGACCGGTTTATAAACTCGCTTGCTGCATCAGACTTGTAGTAGTTGCACCGCATGAGTTTTAGAGCTAG
	FastTrack 3Hr1G090980 Tar3	CTGACCGGTTTATAAACTCGCTTGCTGCATCAGACTTGTCGCCGATGTTGATGACCAGTTTTAGAGCTAG
Nested-PCR	PDS NestFwd	GCAAAATACCTGGCAGATGCTG
	PDS NestRev	CG(A)GAGATCAGTTTGTACTTCA (Golden Promise)
	3Hr1G090980 NestP4F	ATATATCCACTCGCCCACAGC
	3Hr1G090980 NestP4R	AGCTGCTCGCTTGTTAGGAA
	3Hr1G090980 NestP3R	CGGGGATTGGTTCGTGCTG
	3Hr1G090980 NestP5F	AAAGACGAAAGGGACCCTGG
	3Hr1G090980 NestP5R	GCGAACAGACAAAGCGGAGA
	3Hr1G090980 NestP6F	GGTACAAGGTTTCGCCACGG
Sequencing	pBarge final assembly P12F	TGGTGGCAGGATATATTGTGGTG
	pBarge final assembly P12R	GGATAAACCTTTTCACGCCCT
	T Easy T7	TAATACGACTCACTATAGGG
	T Easy Sp6	ATTTAGGTGACACTATAG
